# The predator odor 2,4,5-trimethylthiazoline binds and activates receptor guanylyl cyclase-G to elicit innate defensive responses

**DOI:** 10.1186/2050-6511-16-S1-A40

**Published:** 2015-09-02

**Authors:** Ying-Chi Chao, Ruey-Bing Yang

**Affiliations:** 1 Molecular Medicine Program, Taiwan International Graduate Program; 2 Institute of Biomedical Sciences, Academia Sinica, Taipei, Taiwan; 3 Institute of Pharmacology, School of Medicine, National Yang-Ming University, Taipei, Taiwan

## Background

Guanylyl cyclase (GC)-G is the last member of the receptor GC family [1,2]. Our recent studies demonstrated that GC-G expressed in Grueneberg Ganglion (GG) neurons can be activated by cool temperatures to generate ultrasound calls by isolated pups to elicit maternal care [3]. Detecting the semiochemical warnings present in the environment is essential for species survival. The mouse GG is the olfactory subsystem that also detects alarm pheromones (APs) and other structurally-related chemicals involuntary released by rodent's predators [4,5]. The predator odor 2,4,5-trimethylthiazoline (TMT), a volatile compound originally isolated from the anal secretions of the red fox, induces robust freezing behaviors in mice. TMT shared a similar chemical structure to APs and can activate GG neurons [5]. However, whether TMT can directly bind and stimulate GC-G activity to trigger innate fear responses remains unknown.

## Materials and methods

A combination of biochemical and molecular biology methods, Ca^2+^ imaging as well as behavioural studies comparing wild-type and GC-G-knockout (KO) mice was used to elucidate the molecular and biological function of GC-G in transmitting TMT signaling.

## Results

We show that GC-G can be stimulated by TMT in both in vivo cellular cGMP accumulation assays and in vitro GC assays with isolated GC-G membranes protein. Furthermore, domain deletion analysis verifies that the extracellular domain of GC-G is required for TMT-induced cGMP production. A direct interaction with notable affinity between TMT and GC-G extracellular domain was confirmed by time-resolved surface plasmon resonance. HEK-293T cells co-expressing GC-G and the cGMP-activated ion channel CNGA3 respond to TMT via a rapid influx of calcium. In line with these findings, TMT-induced calcium transients in the GG as well as TMT-evoked innate fear behaviors and an increase of serum corticosterone (a stress hormone) were markedly attenuated in the GC-G-KO mice compared to wild-type littermates.

## Conclusions

Our data demonstrated for the first time that TMT may be a potential ligand for GC-G receptor and unravelled the molecular interaction involved in the inter-specific olfactory message communication between predators and preys via TMT-GC-G signaling.
